# A Review of Frailty Syndrome and Its Physical, Cognitive and Emotional Domains in the Elderly

**DOI:** 10.3390/geriatrics2040036

**Published:** 2017-11-16

**Authors:** Mina Khezrian, Phyo K. Myint, Christopher McNeil, Alison D. Murray

**Affiliations:** 1 Aberdeen Biomedical Imaging Centre, University of Aberdeen, Aberdeen AB25 2ZD, UK; c.mcneil@abdn.ac.uk (C.M.); a.d.murray@abdn.ac.uk (A.D.M.); 2 Institute of Applied Health Sciences, University of Aberdeen, Aberdeen AB25 2ZD, UK; phyo.myint@abdn.ac.uk

**Keywords:** frailty, physical deficit, cognitive impairment, emotional impairment

## Abstract

Background: Frailty, a very important complication of increasing age, is a well-recognised concept although it has not been accurately measured in the clinical setting. The aim of this literature review is to summarise commonly used frailty screening tools, and to describe how new measurement methods have been developed recently. Methods: Several frailty measurement tools including the most cited and newly developed scales have been described in this review. We searched the MEDLINE using the search terms; “frailty score, scale, tool, instrument, index, phenotype” and then summarised selected tools for physical, cognitive, emotional and co-morbidity domains. Results: The most cited frailty measurement methods developed from 1999 to 2005 are primarily criteria for physical frailty (e.g., frailty phenotype). More recently developed tools (e.g., triad of impairment and multidimensional frailty score) consider cognitive and emotional domains in addition to physical deficit in measuring frailty. Co-morbidity has also been considered as a domain of frailty in several measurement tools. Conclusion: Although frailty tools have traditionally assessed physical capability, cognitive and emotional impairment often co-exist in older adults and may have shared origins. Therefore, newer tools which provide a composite measure of frailty may be more relevant for future use.

## 1. Background

Population ageing is a major concern in the 21st century with significant health and socioeconomic consequences. It is estimated there will be more than two billion people globally aged 60 years or over by 2050 [1]. Cumulative decline in many physiological systems and the increased risk of vulnerability resulting from the ageing process is conceptualised as frailty. Frailty is described as one of the most challenging expressions of ageing that decreases the quality of life and independence of older adults [2]. Older frail people also experience dramatic decline in physical and mental functions and have poorer outcomes after even apparently minor stressors such as mild physical disorders and anxiety [3]. Although frailty is a long-established and well-recognised clinical syndrome, there is no consistent way of measuring it in the clinical setting [4]. A model of frailty that could precisely measure and therefore predict this syndrome to potentially prevent, delay its progress, or reverse it at early stages is of great importance in the older adults’ health [5].

It is also recognised that frailty should be seen as a multidimensional syndrome [6] affecting the physical and mental function of the elderly. Cognitive [7,8,9] and emotional impairment [10] resulting from ageing had been frequently reported in the frail older people while, many of the screening tools determine only physical deficits as a proxy measures of frailty [5]. Considering the concurrence of mental and physical dysfunction in the elderly, it seems that there is a necessity of including mental impairment in addition to physical impairment in measuring frailty [11].

Co-existence of multiple chronic conditions or co-morbidity is common in older people. Co-morbidity could negatively affect the physical and mental function of older adults; however, it should be distinguished from the frailty concept due to different prognoses, outcome and treatment strategies [12].

The purpose of this literature review is to summarise the most used frailty measurement methods along with recently developed frailty tools, which incorporate mental function in addition to physical frailty. We selected the six best-known tools based on the recent systematic review by Buta et al. [13]. Frailty tools had been mapped onto to physical, cognitive, emotional and co-morbidity domains.

## 2. Ageing and Frailty

Ageing is associated with a gradual decline in physical functioning. However, the rate of decline varies and hence ageing is not necessarily coupled with frailty [14]. The concept of frailty underpins the need to better understand the variance in individuals with the same chronological age in their function, resilience and adverse outcome [15]. Many protective and risk factors may influence frailty during the life course. Many of these factors have complex interactions among one another as well as independent effects on frailty. Therefore, it is important to understand the onset and progression of frailty in older people in order to develop methods for early detection and prevention. Early life, mid-life and late life predictors and risk factors (e.g., genetic, epigenetic, psychological, socio-economic, lifestyle characteristics, nutritional status, multi-morbidity and medications) should be considered to explain the diversity in population ageing more accurately [5,14].

It is also important to consider the difference between men and women in frailty status in late life. A systematic review reported that at any given age women have a higher Frailty Index score, although, they tolerate the adverse health outcome of frailty better and live longer than men [16]. There are many possible explanations for this phenomenon including differences in inflammatory markers [17], hormones, disease patterns and behavioral heterogeneity [18] in men and women. Studies also suggested that the higher prevalence of frailty in women might be due to psychological symptoms and lower cognitive functioning [18,19].

## 3. Frailty as Physical Impairment

Frailty phenotype is the most well-known frailty assessment developed by Fried et al. in 2001 [20]. This model defines frailty as the existence of three or more of the following five components of physical impairment in older adults: *shrinkage (weight loss), exhaustion, weaknesses, low gait speed* and *low physical activity*. Similarly, impaired physical function has been identified as a surrogate marker of frailty in the Vulnerable Elders Survey (VES-13) [21] and physical frailty measurement tools developed by Gill et al. [22] (Table 1).

These measurement methods consist of a subjective measure of physical activity (self-reported items) as well as directly quantified variables, such as single chair stand test. Loss of mass, strength and function of muscles or sarcopenia is a key component of frailty in the physical impairment models of frailty [20]. Gait speed is another reliable key component of physical frailty models [22].

Some degree of physical dysfunction is an inevitable consequence of ageing and manifests as a key feature in many frail older adults. Therefore, these frailty tools successfully recognize the physical dimension of frailty, while they fail to consider the impact of cognitive and emotional function in development and progression of frailty and its impact on outcomes such as quality of life and independent living. The manifestations of an ageing brain including cognitive and emotional impairment could influence the physical function of older adults. It is well recognised that an impaired mood is associated with reduced physical activity, increase in sarcopenia and therefore increases the prevalence of frailty [29]. There is also growing evidence of a strong association between cognitive decline and physical frailty with shared subcellular pathophysiology that increase the vulnerability that causes a poorer outcome in older adults [9,11,30].

## 4. Frailty as a Co-Morbidity Index

The Frailty Index (FI) and clinical frailty scale developed by Rockwood and Mitnitski from 1999 to 2005 [23,24,25,26] count deficits in health to create a frailty measurement tool. Items include the presence and severity of current diseases, ability in activities of daily living, and physical and neurological signs from the clinical examinations [26]. The FRAIL scale, developed by Abellan van Kan et al. also considers the accumulation of deficits as a substitute measure of frailty. Using this scale, a deficit is recorded for illness if the participant reported more than five from a list of 14 different chronic diseases [27,28] (Table 1).

The relationship between chronic conditions and frailty is complex because they share common risk factors and biological pathways [31]. Multiple chronic diseases (or multi-morbidity) are prevalent in frail older people and can significantly decrease the quality of life [14]. Indeed, frailty is an unavoidable outcome of the advanced stage of some chronic disorders such as arthritis, congestive heart failure and diabetes [31]. Nevertheless, they do not necessarily co-exist; distinguishing between them has important clinical implications because they confer different prognoses, thus specific interventions are required [12].

## 5. Frailty, Cognitive and Emotional Impairment

A comprehensive frailty tool could potentially enable us to better predict adverse outcome and provide a therapeutic target for intervention in the elderly. The requirement to include cognitive and psychological factors in identification, assessment and management of frailty has been increasingly recognised [32,33].

A study by Rothman et al. provided strong evidence to support the use of cognitive impairment as an independent criterion of frailty. They investigated the prognostic effect of seven potential frailty criteria, including five from the Fried phenotype in addition to cognitive impairment and depressive symptoms. The results of this study showed that cognitive impairment was independently associated with chronic disability, long-term nursing home stay, and death [34]. Similarly, a prospective cohort study in patients with a coronary artery disease included cognitive and emotional impairment as a criterion of frailty in addition to physical impairment. This frailty index accurately predicted increased disability and meaningful decline in health-related quality of life at 12 months in this study population [35].

Ageing is associated with the structural and physiological changes in the brain that could affect cognitive and physical function of older adults. Buchman et al. [9] proposed common pathological pathways such as macro infarcts, and Nigral neuronal loss may explain the correlation between cognitive decline and physical frailty. In an earlier study, they also reported that increase in physical frailty is associated with brain pathologies of Alzheimer diseases (AD) and concluded that physical frailty is a non-cognitive manifestation of AD pathology that precedes the onset of dementia [7].

A recent cohort study by Murray et al. showed that hyperintensities in the deep brain structure are linked to depressive symptoms and that this association is mediated via impairment in cognitive and physical function [36]. Furthermore, chronic inflammation that is associated with poor physical function and frailty also plays an important role in vascular cognitive impairment [37]. Recent studies have shown that depression is significantly associated with frailty in older adults and that the two syndromes have common pathophysiological mechanisms such as hormonal changes and inflammation [29].

## 6. Frailty as Multidimensional Impairment 

Recent studied have considered the multidimensional nature of frailty by including cognitive and emotional impairment as critical domains.

Kennedy et al. used a questionnaire to create a frailty index using the Canadian Multicentre Osteoporosis Study (CaMos) dataset. The CaMos Frailty Index (CaMos FI) is based on the cumulative deficit model and includes cognition and emotional function in assessing frailty [38]. CaMos FI quantifies the incidence of fractures over 10 years according to a degree of frailty in men and women aged 25 years and older. However, there is a concern regarding the validity of the responses from cognitively impaired participants (Table 2).

The Multidimensional Frailty Scores (MFS) [39] and the Care Partner- Derived Frailty Index (CP-FI-CGA) [40] were developed based on the comprehensive geriatric assessment (CGA). CGA is a gold standard for management of frail older people, involving a holistic, multidimensional, interdisciplinary assessment of an individual and is associated with improved outcomes in the elderly [42]. Mental impairment, including memory and mood, and chronic conditions are considered as domains of frailty in the CGA based measurement tools (Table 2).

For measuring MFS physical, cognitive and emotional functions are evaluated by the activity of daily living (ADLs) and instrumental ADLs (IADLs), the Korean version of the Mini-Mental State Examination and the Korean Geriatric Depression Scale respectively. MFS also assesses nutritional status, the risk of delirium, co-morbidity and malignant diseases, serum albumin and mid-arm circumferences as well as other domains of frailty. This frailty indicator can independently predict postoperative complications and prolonged hospital stay, however the sample included women only and therefore this study might not be generalizable in the community setting [39]. In addition, CGA based frailty indices have been criticized as cumbersome because they need a trained geriatric team and are time consuming in the clinical setting [42].

CP-FI-CGA has been developed to improve the feasibility of the CGA utilization in developing a frailty index. It is based on a questionnaire including 44 deficit items that are completed by the care partner and applied to predict the survival in 200 patients admitted to the emergency medical services and geriatric ambulatory care. The contribution of carers is notable particularly in the presence of cognitive impairment. Similar to other deficit models, CP-FI-CGA includes chronic conditions as components of frailty [40].

The co-occurrence of physical, cognitive and emotional decline had been collectively named triad of impairment (TOI) and used as a surrogate marker of frailty in the Cardiovascular Health study [43] and Aberdeen Birth Cohort (ABC) study [36,44]. TOI proposed in the ABC study included both subjective (self-reported; SF-36, HADS depression and anxiety questionnaire) and objective (memory tests, gait speed test) measures of function quantifying as a continuing variable to represent frailty status. Using a graded measurement tool will enable a clinician to both identify and grade frailty for severity [2]. This frailty score had been developed in a relatively healthy sample of men and women aged around 64 years and 68 years, it is easy to measure and therefore it is applicable in the clinical setting for general population. Early-life cognitive ability is a strong predictor of TOI in late life and occupational profile, polypharmacy and personality traits such as neuroticism have been shown to be associated with it. This study has revealed the importance of considering a life-course approach to predict and potentially reduce frailty in older adults. However, the validity of TOI and whether it could predict adverse health outcomes such as hospitalization and mortality rate in late life need to be estimated in other studies [44].

In the Rotterdam study, a frailty index had been created based on the accumulation of a deficit model and forty-five different deficits related to mood, cognition, nutritional status, biomarkers, functional status and diseases and conditions have been assessed. The criterion validity of it had been evaluated by investigating the association between the frailty index and survival. Although the Rotterdam FI considers different dimensions of frailty, including cognitive and emotional function, it is time consuming and contains many expensive measurements (such as biomarkers) making it less practical in the clinical setting [41].

## 7. Summary

In this narrative review, we have summarised the most-cited and recently developed frailty tools that include physical, cognitive, emotional and co-morbidity domains and discussed the advantages and drawbacks briefly. The challenge in developing a comprehensive, easily applicable tool to quantify frailty is still ongoing and will always be a compromise between ease of use and complexity of domains assessed. There is increasing recognition of mental frailty as component of frailty tools and this approach has become more common. It is important to recognise that frailty is a spectrum and distinction as frail or not-frail is simplistic, particularly in the context of individual variation in social vulnerability. Analysis of the independent impact of frailty on outcome may help to better understand the effectiveness of frailty prevention or management strategies.

## Figures and Tables

**Table 1:** Most cited frailty measurement tools.

Tools/Study	Authors	Physical Domain/Tests	Emotional Domain/Tests	Cognitive Domain/Tests	Co-Morbidity
Frailty phenotype/Cardiovascular Health Study	Fried et al. 2001 [20]	- Unintentional weight loss (shrinkage) - Self-reported exhaustion - Weakness (grip strength) - Slow walking speed - Low physical activity	No	No	No
Frailty Index (FI; accumulation of deficit)/Canadian Study of Heath and Aging	Rockwood et al. 1999 [23]	- Walk with help - Needing assistance with Activity of Daily Living (ADL)	No	Cognitive impairment	- Bladder or bowel incontinence - Diagnosis of dementia
	Mitnitski et al. 2001 [24]	92 deficits presentation as yes and no including: - Abnormal laboratory values - Disabilities	- Difficulty with mood - Feeling sad or depressed etc.	Difficulty with memory etc.	- Diabetes - Stroke etc.
	Mitnitski et al. 2002 [25]	20 deficits presentation as yes and no including: - Impaired mobility - Gait abnormality - Impaired vibration sense - Difficulty in toileting, cooking, bathing, going out, grooming, dressing - Changes in sleep - Limb tone abnormality	No	No	- Vision loss - Hearing loss - Vascular problems - Resting tremor - Diabetes - Hypertension - Urinary complaints - Skin problems - Gastro-intestinal problem
Clinical Frailty Scale continue of the FI/Canadian Study of Heath and Aging	Rockwood et al. 2005 [26]	70 deficits presentation as yes and no including: - Abnormal laboratory values - Disabilities - Falls	- Mood problems - Feeling sad, blue, depressed - History of depressed mood	- Memory changes - Short-term memory impairment - Long-term memory impairment	- Cardiac problems - Myocardial infarction - Arrhythmia - Congestive heart failure
The Vulnerable Elders Survey (VES-13)	Saliba et al. 2001 [21]	- Age - Self-rated health - Self-reported physical function limitation in walking, bending, reaching etc. - Needing assistance with Activity of Daily Living/Instrumental Activity of Daily Living	No	No	No
Physical frailty/Randomised controlled trail	Gill et al. 2002 [22]	- Rapid gait test (walking back and forth over a 10-foot (3-m) course as quickly as possible) - Single chair stand	No	No	No
Frail scale/Health in Men Study	Abellan van kan et al. 2008, Hyde et al. 2010 [27,28]	- Fatigue (SF-36) - Resistance—ability to climb a single flight of stairs (SF-36) - Ambulation—ability to walk one block (SF-36) - Loss of weight—more than 5% (between 4 to 5 years)	No	No	- Illnesses more than 5 in list of 14 diseases including: arthritis, diabetes, dementia, angina or myocardial infarction, hypertension, stroke, asthma

**Table 2:** Recently developed frailty measurement tools.

Tools/Study	Authors	Physical Domain/Tests	Emotional Domain/Tests	Cognitive Domain/Tests	Co-Morbidity
CaMos Frailty index/Canadian Multicentre Osteoporosis Study	Kennedy et al. 2014 [38]	30 deficit items including: - Walking - General health - Limitation in lifting/carrying groceries etc.	- Interference with social activities due to physical/emotional health (last 4 weeks)	- Cognition in six levels: able to remember most things, think clearly and solve day to day problems = 0 to unable to remember anything at all and unable to think or solve day to day problems = 1	- Osteoarthritis - Breast cancer - Hypertension etc.
Multidimensional Frailty Score (MFS)	Choi et al. 2015 [39]	- Serum albumin - Mid-arm circumference - Activity of Daily Living (modified Barthel Index) - Instrumental Activity of Daily Living (Lawton and Brody Index) - Nutritional status (Mini Nutritional Assessment)	- Depression (short form of the Korea Geriatric Depression Scale)	- Dementia (Korean version of the Mini-Mental State Examination) - Delirium(Nursing Delirium Screening Scale)	- Charlson Comorbidity Index - Malignant disease
Care Partner-derived Frailty Index based upon Comprehensive Geriatric Assessment (CP-FI-CGA)	Goldstein et al. 2015 [40]	Yes or no answer to the following questions: - Falls - Sleep problems - Exhaustion - Speech problems - Loss of appetite - Balance problems - Dizzy or lightheaded - Assistance with walking (aid, stand by) - Hold onto furniture to prevent falls - Difficulties in getting out of bed or chair, walking, managing medications… - Unable to Drive - Weight Loss (more than 10 pounds in six months) - Weakness	- Depression Yes = 1, Low Mood = 0.5, No = 0 - Anxiety - Health attitude Excellent = 0, Good = 0.25, Fair = 0.5, Poor = 1.0	-Memory Problems	- Hypertension - Stroke - Diabetes Arthritis Parkinson’s Disease, recent broken bones, Problems including: Heart, Teeth, Lungs or breathing, stomach, kidneys, feet, skin, thyroid, hearing, eyesight, bowel, bladder
Triad of impairment (TOI)/Aberdeen Birth Cohort	Murray et al. 2016 [36]	- Subjective measure of physical health (SF-36) including: Physical Functioning, Role-Physical Bodily Pain and General Health - Walks time (the time in seconds for walking 6 meters normalised for height)	- Self-reported Hospital Anxiety and Depression Scale (HADS-A-D) - Subjective measure of emotional health (SF-36) including: Vitality, social functioning, role-emotional and mental health	Memory tests including: - Non-verbal reasoning (Raven’s Standardised Progressive Matrices) - Spatial ability (Block Design Test) - Mental speed (Digit Symbol Test) - Verbal memory (Rey’s Auditory Verbal Learning Test)	No
Frailty Index (FI)/Rotterdam study	Schoufour et al. 2016 [41]	- BMI - Biomarkers - Falling - Joint complaints - Mobility - Stanford Health Assessment Questionnaire for physical activity Activities of Daily Living (Lawton Index) - Hospital admission	- Self-reported Centre for Epidemiologic Studies Depression Scale to measure (CES-D): - Depressed affect - Positive affect - Interpersonal - Somatic and retarded activity	- Forgetfulness - Mini Mental State Examination - Word Fluency test - Stroop test - Aphasia - Letter-Digit Substitution Test: the number of correct digits	- Hyperlipidaemia - HDL - Systolic blood pressure - Cancer - COPD/Asthma - Cardiovascular diseases - Stroke - Diabetes Mellitus - Age-related macular degeneration
